# Quality of life in patients with type 2 diabetes after switching to insulin degludec: results from a cross-sectional survey

**DOI:** 10.1007/s11136-020-02753-6

**Published:** 2021-02-07

**Authors:** Chioma Uzoigwe, Michael Radin, Carol M. Hamersky, Mitch DeKoven, Cassie Holt, Swapna Karkare, William H. Polonsky

**Affiliations:** 1 Novo Nordisk Inc., 800 Scudders Mill Road, Plainsboro, NJ 08536 USA; 2 IQVIA, Falls Church, VA USA; 3 IQVIA, New York, NY USA; 4 IQVIA, Deerfield, IL USA; 5 Behavioral Diabetes Institute, San Diego, CA USA; 6 University of California, San Diego, CA USA

**Keywords:** Quality of life, Type 2 diabetes, Basal insulin, Insulin degludec, Insulin glargine, Hypoglycemia

## Abstract

**Purpose:**

Five quality of life (QoL) domains are particularly important to patients with type 2 diabetes (T2D) using basal insulin—sense of physical well-being, sense of safety regarding hypoglycemia, sense of diabetes as burdensome, feelings of freedom and flexibility, and sleep quality.

**Methods:**

An online survey assessed these QoL domains in adult patients with T2D in the USA who had switched from a previous basal insulin to insulin degludec (IDeg): modified versions of the World Health Organization (Five) Well-Being Index (WHO-5), Hypoglycemia Attitudes and Behavior Scale (HABS; confidence and anxiety subscales only), and Diabetes Distress Scale (DDS; emotional burden and regimen-related distress subscales only); three items assessing feelings of freedom and flexibility; and one item assessing sleep quality (hours of restful sleep). Patients rated each item for their previous basal insulin and currently while using IDeg. Correlations between sleep quality and the other QoL scales were also assessed.

**Results:**

In total, 152 patients completed the survey and were included in the study sample. Patients reported significantly improved scores while using IDeg on all WHO-5, DDS, HABS, feelings of freedom and flexibility item scores, and total raw/mean subscale scores (*P* < 0.0001). Patients also reported a significantly greater number of hours of restful sleep [mean (*SD*) 6.6 (2.0) vs. 5.5 (1.8); *P* < 0.0001]. Better sleep quality statistically significantly correlated with improved QoL in all other domains assessed.

**Conclusions:**

Treatment with IDeg after switching from a previous basal insulin was associated with statistically significant improvements in all QoL domains assessed.

**Supplementary Information:**

The online version contains supplementary material available at 10.1007/s11136-020-02753-6.

## Introduction

The addition of basal insulin is a well-established approach in patients with type 2 diabetes (T2D) who require intensification of antihyperglycemic therapy [1, 2]. Insulin degludec (IDeg) is one of several second-generation long-acting insulin analogs approved in the USA to improve glycemic control in patients with diabetes mellitus, available in the USA as of January 2016 [3, 4]. IDeg has a long duration of action and offers the convenience of once-daily dosing at any time of day [3, 5]. Studies of the blood glucose-lowering effect of IDeg have shown that IDeg has a flatter, more stable pharmacodynamic profile compared with insulin glargine U-100 and U-300 [5, 6]. In addition, randomized controlled trials have found that IDeg is associated with significantly lower rates of severe hypoglycemia and nocturnal hypoglycemia than is insulin glargine U-100 in patients with T2D while providing equivalent glycemic control [7–9]. However, success of antihyperglycemic treatments in real-world clinical practice is determined not only by their clinical effectiveness and safety profile, but also by a range of additional factors that may affect treatment satisfaction and, hence, adherence [10]. Therefore, to obtain a comprehensive understanding of the potential value of a diabetes intervention, it is important to evaluate treatment outcomes in the broader context of treatment-related factors that may impact quality of life (QoL) [10].

The impact of IDeg on QoL in patients with T2D was previously assessed in a meta-analysis of three clinical trials of 26- or 52-weeks’ duration in which patients were randomized to receive IDeg or insulin glargine U-100 [11]. Compared with insulin glargine U-100, IDeg improved both mental and physical health status on the widely used generic 36-item Short Form (SF-36) questionnaire. Anecdotal evidence has suggested that treatment with IDeg may be associated with unique and potentially important QoL benefits, which are often reported by patients simply as “feeling better” [12]. These findings are supported by a qualitative study that was conducted in patients with T2D who switched to IDeg from another basal insulin [12]. In this study, four major factors were identified that contributed to patients’ sense of “feeling better”: a reduced sense of diabetes as burdensome and requiring excessive attention; enhanced feelings of adaptability and freedom; increased sense of security, particularly around concerns related to hypoglycemia; and a greater sense of physical well-being [12]. A review of the literature and of patient blogs [13–17] led to the identification of one additional domain, sleep quality, which appears to influence QoL.

To further explore and validate these observations, we sought to determine in a more quantitative fashion the unique domains related to the experience of “feeling better” when using IDeg, as compared with the patient’s previous basal insulin. Five QoL domains were examined: three were diabetes-specific (perceptions of safety with regards to hypoglycemia, sense of diabetes as burdensome, feelings of freedom and flexibility in regard to diabetes management) and two were generic (well-being and sleep quality).

The primary aim of this study was to examine the impact between QoL scores with switching from previous basal insulin use to the use of IDeg, and if this was affected by the nature of the reason for switching, i.e., if it was primarily due to lack of health insurance coverage for the previous insulin. Secondary aims included exploring the predictors of improved QoL after switching to IDeg and the correlation of sleep quality with other patient-reported outcome (PRO) measures.

## Methods

### Study design and population

This study was a single-assessment, cross-sectional, online survey of patients with T2D in the USA who were taking IDeg. Eligible patients were aged ≥ 18 years, had been taking IDeg for ≥ 3 months, had previously used at least one other basal insulin before taking IDeg, and had first started taking insulin ≥ 2 years after being diagnosed with T2D. Patients were identified from a commercially available diabetes patient panel and were invited by email to participate. A target of collecting at least 150 completed surveys was set based on statistical power calculations using 2017 T2D prevalence rates, to permit subgroup analyses, and to ensure a diverse patient sample.

Respondents were asked to provide written informed consent and to complete a short eligibility questionnaire. All respondents who met the eligibility criteria and completed the survey were included in the study analyses. For participation in this study, they received an honorarium of US$10 in the form of reward points that were redeemable for a gift card or cash. Confidentiality and privacy of the study data were managed in accordance with Health Insurance Portability and Accountability Act of 1996 regulations, and all study analyses used anonymized data stored on secure servers. Ethics approval of the study protocol was obtained from New England Independent Review Board. As this was a non-interventional study, trial registration was not required.

### Survey questions

The survey instrument was developed jointly by IQVIA and Novo Nordisk, Inc. (Plainsboro, NJ, USA); it was administered by Nielsen Holdings, Inc. (New York, NY, USA) from March 29 to May 7, 2018. The survey was developed by first performing a focused literature review to identify generic or diabetes-specific PRO instruments that mapped to the domains of interest in this study. IQVIA and Novo Nordisk, Inc. then collaborated with a diabetes psychologist (WHP) to judge the clarity, relevance, value, and priority of the items selected.

The survey consisted of 70 questions and took approximately 20 min to complete. To identify any potential issues, the content was reviewed by two eligible patients. The survey’s structure and scoring scheme are summarized in Online Resource 1. Data on demographics and clinical history were obtained both from the eligibility questionnaire and from 12 of the questions in the main survey itself. The remaining 58 questions in the survey asked patients about the five QoL domains that were the focus of this study.

To allow comparison with previous studies, the domains of “well-being” and “sense of diabetes as burdensome” were assessed using modified versions of the World Health Organization (Five) Well-Being Index (WHO-5) and Diabetes Distress Scale (DDS), respectively, as versions of these instruments had previously been used to assess these domains in a large online survey of patients with type 1 diabetes (T1D) [18]. The WHO-5 is a commonly used five-item generic instrument that has been validated as a measure of well-being in patients with T1D and T2D [19, 20]. A change of 10 points in WHO-5 total percentage score is considered to be clinically relevant [21, 22]. The DDS comprises four subscales (emotional burden, physician-related distress, regimen-related distress, and interpersonal distress) and has been used both as an outcome measure in several clinical studies, and as an instrument to facilitate communication with patients in clinical practice [23]. This instrument is a validated measure of diabetes-related emotional distress for both T1D and T2D populations [24, 25]. Only the emotional burden and regimen-related distress subscales were included in the present study.

“Sense of safety with regards to hypoglycemia” was assessed using a modified version of the Hypoglycemia Attitudes and Behavior Scale (HABS). This instrument consists of three subscales (avoidance, confidence, and anxiety) and has been validated for use in adults with T2D [26, 27]. In our study, we included only the confidence and anxiety subscales.

The standard versions of the WHO-5, DDS, and HABS all ask patients to rate how they feel about their well-being, diabetes-related distress, or hypoglycemia, respectively, during a single specified time period—“over the last 2 weeks” (WHO-5), “during the past month” (DDS), or “current feelings” (HABS) [21, 23, 26]. We modified these three instruments so that patients rated each item twice. First, we asked patients to think back to when they were on their previous insulin (directly before switching to IDeg) and to indicate how they felt at that time. Second, we asked them to indicate how they had been feeling over the last 2 weeks (WHO-5), during the past month (DDS), or at the current time (HABS) while using IDeg.

Our review of the literature did not yield any diabetes-specific PRO measure that could map to “feelings of freedom and flexibility.” Therefore, we assessed this domain using newly generated questions that were designed based on patients’ feedback in the previous qualitative study that investigated QoL in patients who switched to IDeg from another basal insulin [12]. Patients were asked to indicate the degree to which they agreed or disagreed with the three following statements, thinking back to when they were on their previous basal insulin and while using IDeg: (1) feel pressured to eat snacks to avoid low blood sugar problems; (2) feel restricted about if and/or when I can exercise; (3) feel like I can’t be as spontaneous in my life as I want to be.

Each item in the modified WHO-5, HABS, and DDS, and in the “feelings of freedom and flexibility” domain was assessed using either a five- or six-point Likert-type scale (see Online Resource 1). Sleep quality was quantified by asking patients their average number of hours of restful sleep, thinking back to when they were on their previous basal insulin and while using IDeg.

### Statistical analysis

Descriptive statistics were used to characterize the data. Categorical measures were reported using frequency (number of cases) and percentage of the total number of patients observed in each category. Continuous measures were reported using mean, median, standard deviation (SD), and range. Analyses of PRO scores were conducted in the whole study population and in patient subgroups defined according to whether switching to IDeg was due to the previous basal insulin no longer being covered by the patient’s insurance company. For comparative analyses (overall and subgroup), statistical significance was determined by the Wilcoxon signed-rank test for continuous variables. A *P*-value < 0.05 was considered statistically significant. Imputations or substitutions were not performed for missing data.

Both univariate and multivariable linear regression analyses were performed to identify whether any of five key variables (age, sex, ethnicity, T2D duration, and length of time on IDeg) were associated with the difference in scores (defined as score while using the previous basal insulin minus the score while using IDeg) for each of the PRO scales. These five particular variables were significantly associated with the outcomes based on the univariate analysis and served as the independent variables in each multivariable model assessed. In addition, Pearson correlation coefficients were used to assess the correlation between the difference in the average number of hours of restful sleep and the difference in scores on each of the other PRO scales, as we wondered whether improved sleep might have resulted from reductions in hypoglycemia concerns.

## Results

A total of 6378 patients were screened for eligibility, and 152 (2.4%) met the eligibility criteria and completed the survey. As IDeg was new to the market, a large number of patients needed to be screened to reach the target sample size. The most common reasons for not meeting eligibility criteria were that the patient had started taking insulin < 2 years after being diagnosed with T2D or that the patient was not currently taking IDeg. The study population ranged in age from 18 to 86 years, had a mean (*SD*) age of 48.6 (15.7) years, and included a similar number of female (*n* = 78; 51.3%) and male patients (*n* = 74; 48.7%; Table 1). The majority of the sample (55.3%) reported a T2D duration ≥ 6 years. The most commonly used previous basal insulins were insulin glargine U-100 and insulin NPH (Table 1). Approximately two-thirds of patients had been using IDeg for 7 months or longer.

**Table 1:** Patient demographics and clinical characteristics

Characteristic	Total study population (*N* = 152)
Age, years	
Mean ± SD	48.6 ± 15.7
Median (range)	46.0 (18–86)
Age group, *n* (%)	
18–39 years	55 (36.2)
40–64 years	65 (42.8)
≥ 65 years	32 (21.1)
Sex, *n* (%)	
Female	78 (51.3)
Male	74 (48.7)
Ethnicity, *n* (%)^a^	
White	110 (72.4)
Hispanic, Latino, or Spanish origin	19 (12.5)
Black or African American	19 (12.5)
Asian	6 (3.9)
American Indian or Alaskan Native	3 (2.0)
Middle Eastern or North African	2 (1.3)
Native Hawaiian or other Pacific Islander	1 (0.7)
US region, *n* (%)	
South	69 (45.4)
Midwest	35 (23.0)
Northeast	25 (16.4)
West	23 (15.1)
Employment status, *n* (%)	
Employed full-time	79 (52.0)
Retired	43 (28.3)
Employed part-time	12 (7.9)
Homemaker	12 (7.9)
Other	6 (3.9)
Highest degree of education, *n* (%)	
Less than high school diploma	2 (1.3)
High school graduate (diploma or GED)	31 (20.4)
Some college or associate degree	43 (28.3)
Bachelor’s degree	46 (30.3)
Some graduate school	7 (4.6)
Master’s degree or higher	23 (15.1)
Type of health insurance, *n* (%)^a^	
Insurance coverage through a current or former employer	71 (46.7)
Medicare	41 (27.0)
Individual/family insurance plans/Healthcare.gov/Affordable Care Act (i.e., coverage purchased directly by patient)	23 (15.1)
Insurance coverage through spouse’s employer	16 (10.5)
Medicaid (MediCal for California residents)	14 (9.2)
Other	10 (6.6)
Not sure	2 (1.3)
Current marital/relationship status, *n* (%)	
Married	94 (61.8)
Single, never married	36 (23.7)
Widowed	13 (8.6)
Separated	5 (3.3)
Living with partner	4 (2.6)
T2D duration, *n* (%)	
< 3 years	11 (7.2)
3–5 years	57 (37.5)
6–10 years	39 (25.7)
11–15 years	22 (14.5)
≥ 16 years	23 (15.1)
Medical conditions diagnosed prior to using a basal insulin (self-reported), *n* (%)^a^	
High blood pressure	81 (53.3)
High cholesterol or triglycerides	77 (50.7)
Obesity	55 (36.2)
Depression	48 (31.6)
Anxiety	43 (28.3)
None of the above	27 (17.8)
Prior basal insulin, *n* (%)^a^	
Insulin glargine U-100 (Lantus®)	64 (42.1)
Insulin NPH	50 (32.9)
Insulin detemir	42 (27.6)
Insulin glargine U-300	35 (23.0)
Other	7 (4.6)
Switch to IDeg because previous basal insulin no longer covered by patient’s insurance company, *n* (%)	
Yes	43 (28.3)
No	108 (71.1)
Don’t know	1 (0.7)
IDeg duration, *n* (%)	
3–6 months	50 (32.9)
7–12 months	48 (31.6)
> 12 months	54 (35.5)
IDeg concentration, *n* (%)	
U-100	82 (53.9)
U-200	68 (44.7)
Don’t know	2 (1.3)
Units of IDeg taken each day^b^	
Mean ± SD	33.1 ± 41.7
Median (range)	8.5 (1–160)
Last HbA1c level, *n* (%)	
≤ 5.0%	0 (0)
5.1–6.0%	7 (4.6)
6.1–7.0%	41 (27.0)
7.1–8.0%	45 (29.6)
8.1–9.0%	21 (13.8)
9.1–10.0%	15 (9.9)
10.1–11.0%	7 (4.6)
11.1–12.0%	6 (3.9)
≥ 12.1%	0 (0)
Don’t know	10 (6.6)
Blood sugar test frequency (over last month), *n* (%)	
Less than every day	11 (7.2)
Daily	58 (38.2)
Twice a day	40 (26.3)
Three times a day	35 (23.0)
More than three times a day	8 (5.3)

*GED* general education development, *HbA1c* glycated hemoglobin, *IDeg* insulin degludec, *NPH* neutral protamine Hagedorn, *SD* standard deviation, *T2D* type 2 diabetes

^a^Patients could select more than one option, so the percentages sum to more than 100%

^b^*N* = 142

### Impact on QoL after switching to IDeg

**Fig. 1:**
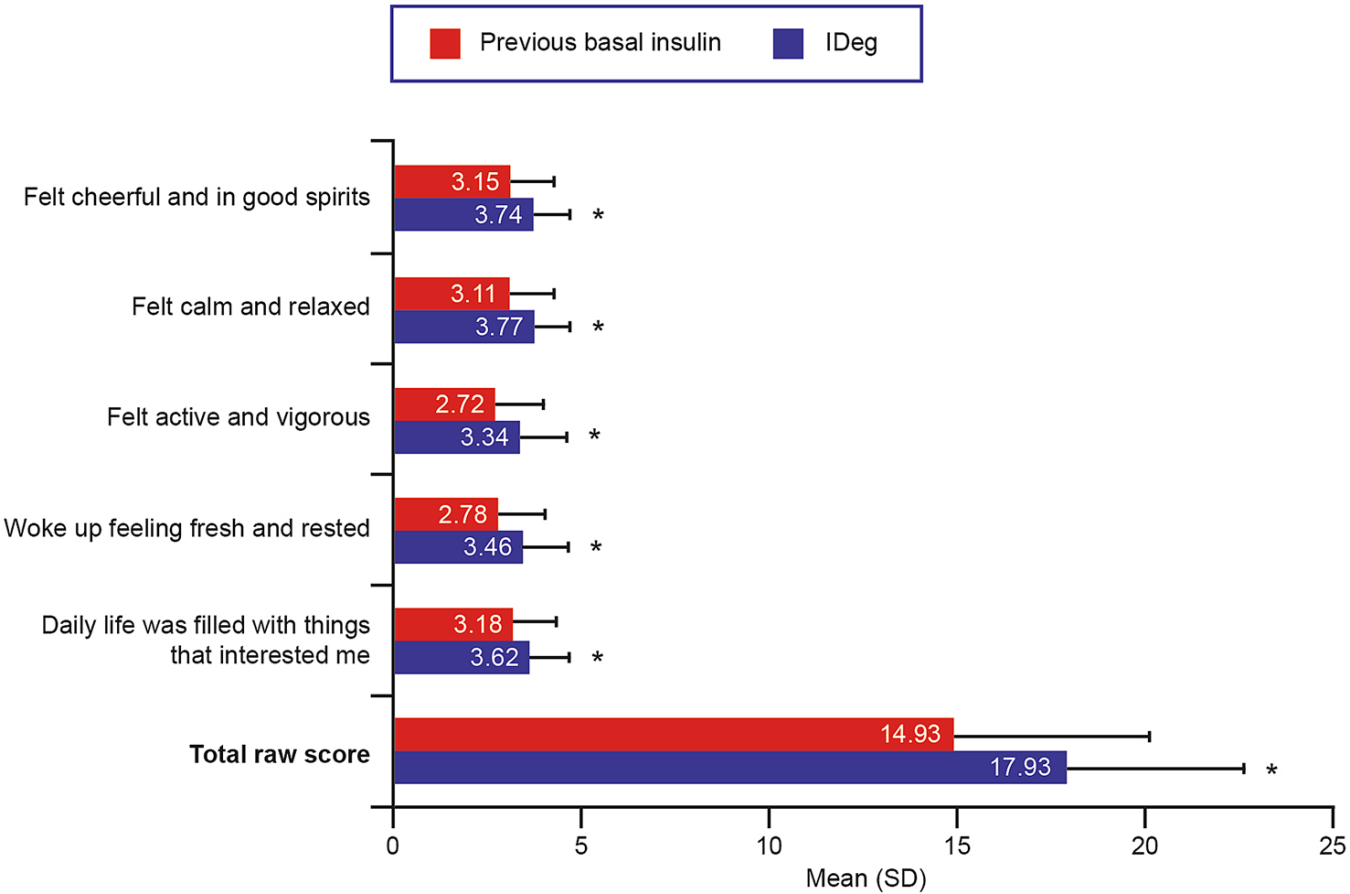
WHO-5 item and total raw scores (*N* = 152). Higher scores indicate better QoL. Total raw score is the sum of the 5-item scores. *P < 0.0001 for score while using IDeg vs. score while using previous basal insulin. *IDeg* insulin degludec, *QoL* quality of life, *SD* standard deviation, *WHO-5* World Health Organization (Five) Well-Being Index

**Fig. 2:**
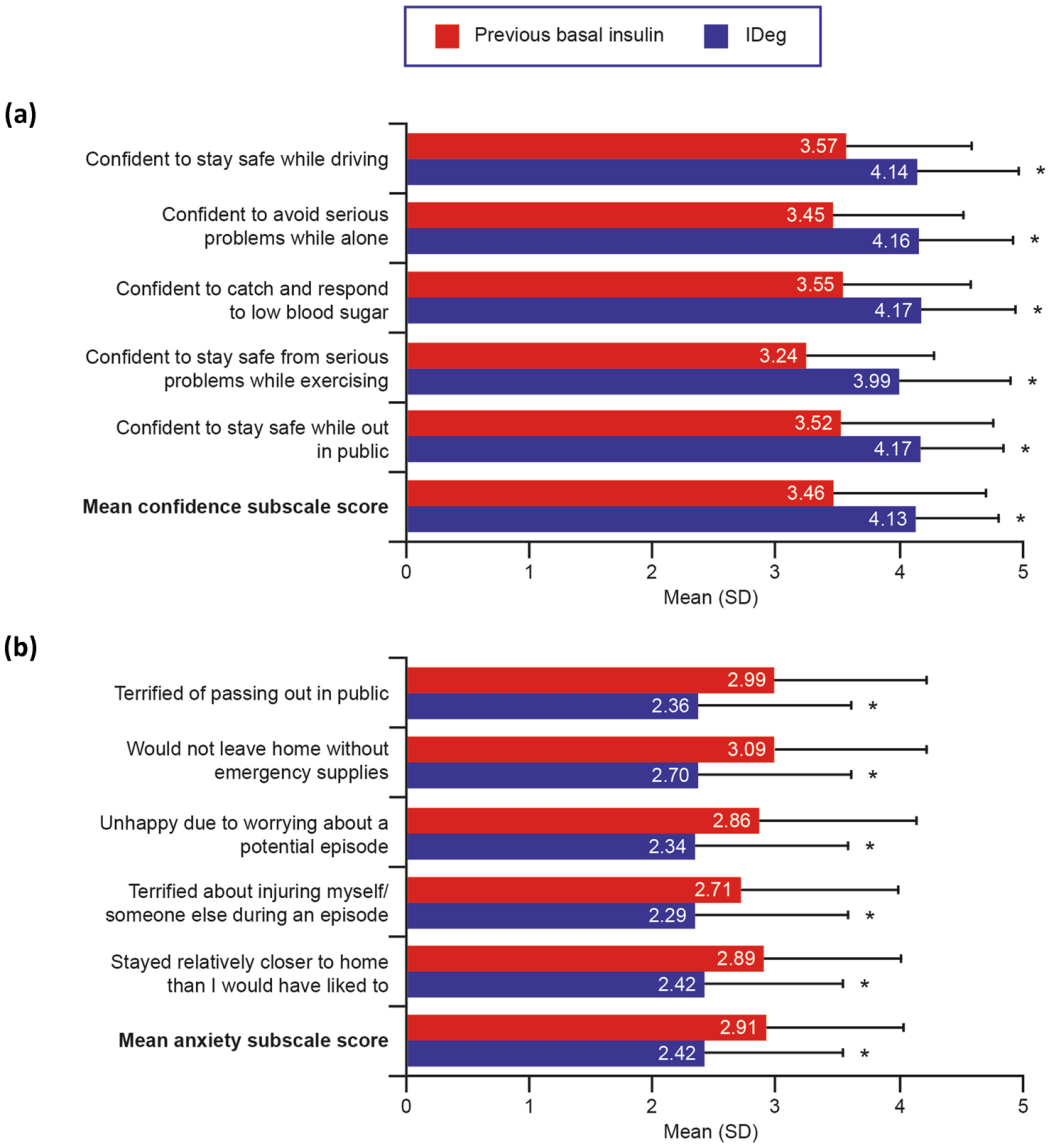
HABS item and mean subscale scores (*N* = 152). **a** On the confidence subscale, higher scores indicate better QoL (i.e., greater confidence). **b** On the anxiety subscale, lower scores indicate better quality of life (i.e., less anxiety). * *P* < 0.0001 for score while using IDeg vs. score while using previous basal insulin. *HABS* Hypoglycemia Attitudes and Behavior Scale, *IDeg* insulin degludec, *QoL* quality of life, *SD* standard deviation

**Fig. 3:**
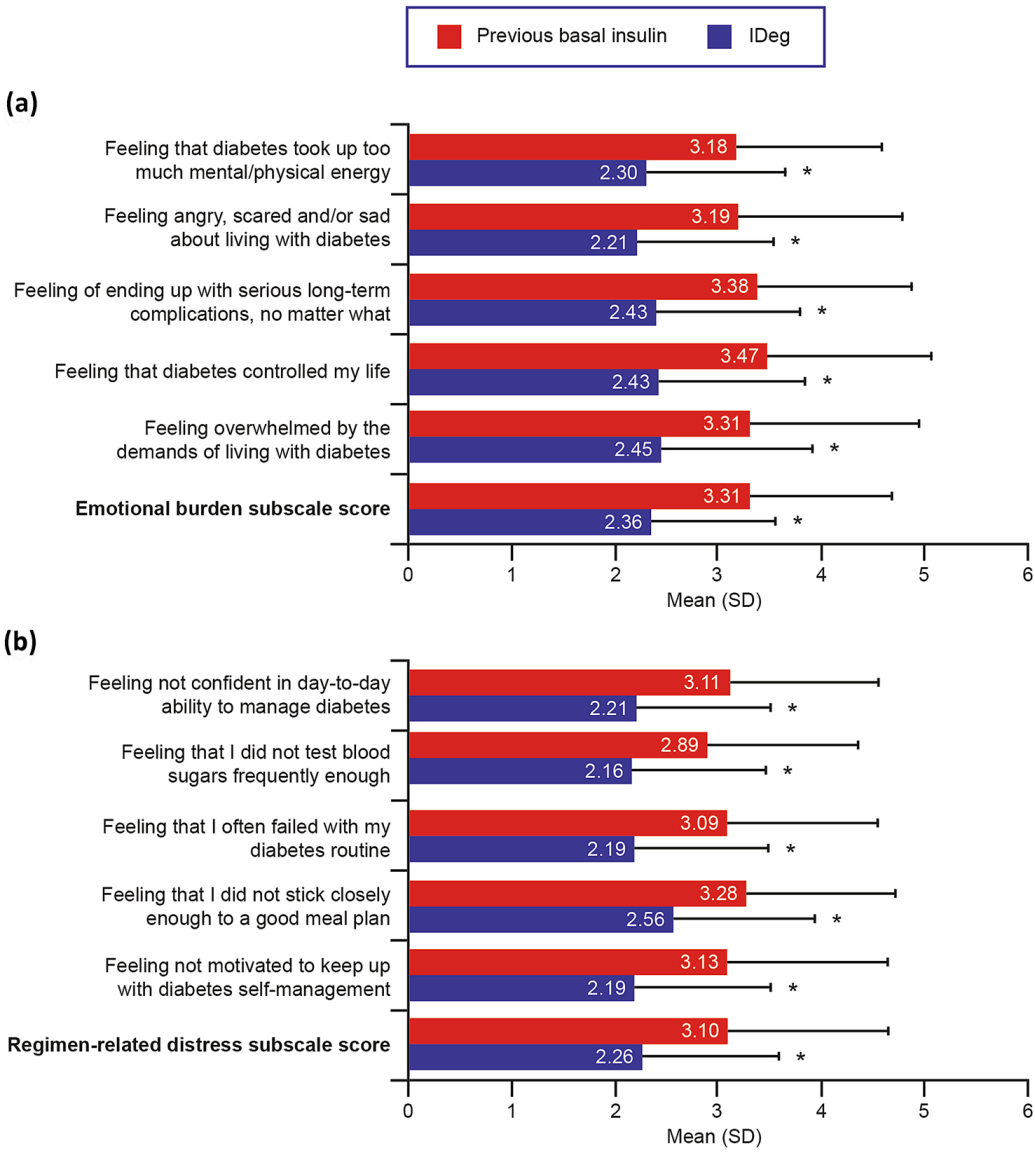
DDS item and mean subscale scores (*N* = 152). On both the emotional burden subscale (**a**) and the regimen-related distress subscale (**b**), lower scores indicate better QoL (i.e., less burden and lower distress). * *P* < 0.0001 for score while using IDeg vs. score while using previous basal insulin. *DDS* Diabetes Distress Scale, *IDeg* insulin degludec, *QoL* quality of life, *SD* standard deviation

Patients reported statistically significantly higher scores on all five WHO-5 items and on the WHO-5 total raw score while using IDeg compared with their previous basal insulin (all *P* < 0.0001; Fig. 1). The mean improvement in WHO-5 total raw score was 3.0 points, equivalent to an improvement of 12.0 points in WHO-5 total percentage score. On the HABS confidence subscale, all item scores, as well as the mean subscale score, were also significantly higher while using IDeg than with the previous basal insulin (all *P* < 0.0001; Fig. 2a). On the HABS anxiety subscale (Fig. 2b), DDS emotional burden subscale (Fig. 3a), and DDS regimen-related distress subscale (Fig. 3b), all item scores as well as the mean subscale score were significantly lower while using IDeg than with the previous basal insulin (all *P* < 0.0001). Improved QoL after switching to IDeg was reported by ≥ 50% of patients on the WHO-5, HABS confidence and anxiety subscales, and DDS emotional burden and regimen-related distress subscales (Online Resource 2).

**Fig. 4:**
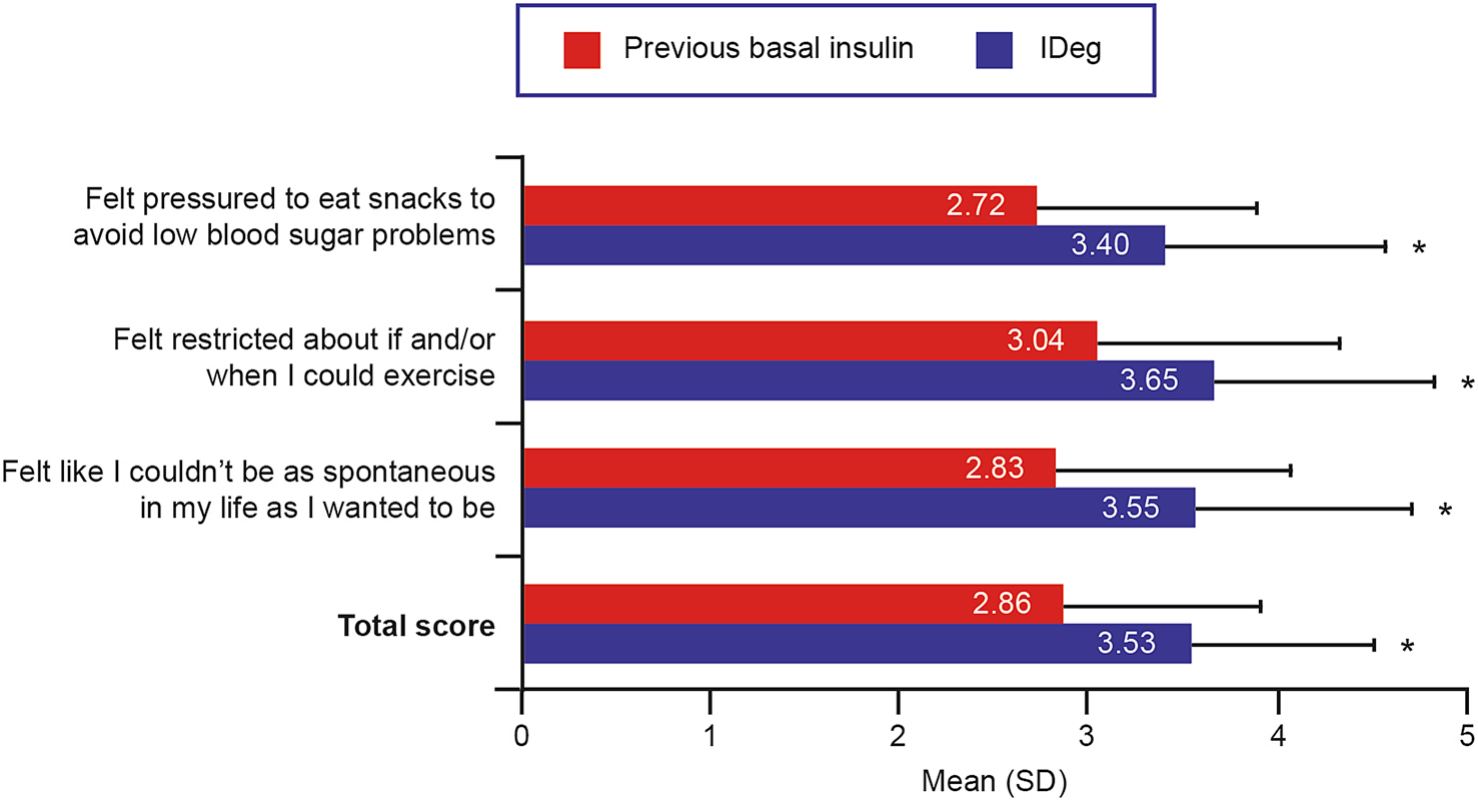
Feelings of freedom and flexibility: item and total scores (*N* = 152). Higher scores indicate better QoL. * *P* < 0.0001 for score while using IDeg vs. score while using previous basal insulin. *IDeg* insulin degludec, *QoL* quality of life, *SD* standard deviation

Scores on all three items assessing patients’ feelings of freedom and flexibility were significantly higher while using IDeg compared with the previous basal insulin (all *P* < 0.0001; Fig. 4). Patients also reported a significantly greater average number of hours of restful sleep while using IDeg compared with their previous basal insulin [mean 6.6 (*SD* 2.0) vs. 5.5 (*SD* 1.8); *P* < 0.0001]. An increased number of hours of restful sleep after switching to IDeg was reported by the majority of patients (59.2%; Online Resource 2).

Patients who were involuntarily switched to IDeg (i.e., previous basal insulin was no longer covered by insurance; *N* = 43) reported significant improvements in HABS anxiety subscale score (*P* = 0.043), DDS emotional burden subscale score (*P* = 0.023), feelings of freedom and flexibility total score (*P* = 0.0039), and average number of hours of restful sleep (*P* = 0.0002; Table 2). Patients who did not switch due to insurance changes (*N* = 108) reported significant improvements in all QoL domains (Table 2; all *P* < 0.0001).

**Table 2:** PRO scores in patients based on insurance-determined basal insulin coverage

PRO score, mean ± *SD*	Switched to IDeg because previous basal insulin was no longer covered by the patient’s insurance company					
	Yes (*N* = 43)			No (*N* = 108)		
	Previous basal insulin	IDeg	*P*-value^a^	Previous basal insulin	IDeg	*P*-value^a^
WHO-5 total raw score	17.00 ± 4.24	17.35 ± 4.31	0.4887	14.10 ± 5.28	18.19 ± 4.83	< 0.0001
HABS mean confidence subscale score	3.71 ± 0.72	3.89 ± 0.66	0.1202	3.37 ± 0.96	4.22 ± 0.65	< 0.0001
HABS mean anxiety subscale score	2.96 ± 1.11	2.80 ± 1.00	0.043	2.89 ± 1.14	2.26 ± 1.13	< 0.0001
DDS mean emotional burden subscale score	3.24 ± 1.16	2.92 ± 1.17	0.023	3.34 ± 1.47	2.14 ± 1.17	< 0.0001
DDS mean regimen-related distress subscale score	3.05 ± 1.09	2.74 ± 1.18	0.3328	3.12 ± 1.29	2.08 ± 1.09	< 0.0001
Feelings of freedom and flexibility total score	2.78 ± 0.98	3.25 ± 0.86	0.0039	2.90 ± 1.07	3.65 ± 1.02	< 0.0001
Average number of hours of restful sleep	5.40 ± 2.12	6.37 ± 2.43	0.0002	5.55 ± 1.71	6.69 ± 1.73	< 0.0001

*DDS* Diabetes Distress Scale; *HABS* Hypoglycemia Attitudes and Behavior Scale; *IDeg* insulin degludec; *PRO* patient-reported outcome; *SD* standard deviation; *WHO-5* World Health Organization (Five) Well-Being Index

^a^*P*-value for PRO score while using IDeg vs. score while using the previous basal insulin

### Predictors of improved QoL after switching to IDeg

The univariate regression analyses found that a T2D duration of < 3 years, non-white ethnicity, and younger age were statistically significant predictors of a greater level of improvement in QoL in multiple domains after switching to IDeg. In the multivariable regression analyses, a T2D duration of < 3 years and non-white ethnicity remained statistically significant predictors of a greater level of improvement in QoL; however, these varied by QoL domain.

### Correlation of sleep quality with other PRO scales

A greater amount of restful sleep was found to be significantly correlated with improved QoL as assessed by WHO-5 total raw score, HABS and DDS mean subscale scores, and each item in the feelings of freedom and flexibility domain (Table 3).

**Table 3:** Correlation between sleep quality and PRO scales

	Correlation coefficient^a^	*P-*value
Sense of physical well-being (WHO-5)	0.40^b^	< 0.0001
Sense of safety (HABS)		
Confidence subscale	0.54^b^	< 0.0001
Anxiety subscale	− 0.32^c^	< 0.0001
Sense of diabetes as burdensome (DDS)		
Emotional burden subscale	− 0.55^c^	< 0.0001
Regimen-related distress subscale	− 0.53^c^	< 0.0001
Feelings of freedom and flexibility		
Felt pressured to eat snacks to avoid low blood sugar problems	0.28^b^	0.0004
Felt restricted about if and/or when I could exercise	0.29^b^	0.0002
Felt like I couldn’t be as spontaneous in my life as I wanted to be	0.37^b^	< 0.0001

*DDS* Diabetes Distress Scale, *HABS* Hypoglycemia Attitudes and Behavior Scale, *IDeg* insulin degludec, *PRO* patient-reported outcomes, *WHO-5* World Health Organization (five) Well-Being Index

^a^Correlation coefficients show the correlation between the difference in the average number of hours of restful sleep (i.e., number of hours while using the previous basal insulin minus number of hours while using IDeg) and the difference in PRO scores (i.e., score while using the previous basal insulin minus score while using IDeg)

^b^On the WHO-5 and HABS confidence subscales, and “feelings of freedom and flexibility” scale, a positive correlation coefficient indicates that a greater amount of restful sleep correlates with better quality of life

^c^On the HABS anxiety subscale and both DDS subscales, a *negative* correlation coefficient indicates that a greater amount of restful sleep correlates with better quality of life (i.e., less anxiety/distress)

## Discussion

Guidelines from the American Association of Clinical Endocrinologists/American College of Endocrinology [2] and American Diabetes Association [28] stress that the choice of insulin therapy should be a collaborative decision between the healthcare provider and the patient. Therefore, given the importance of the patient’s voice, we conducted the present study to quantitatively assess change in QoL across a broad range of domains that previous research highlighted as being of importance to patients. In particular, anecdotal evidence has indicated that patients with T2D often report “feeling better” when treated with IDeg. We therefore sought to understand what this interesting finding might refer to and how we might assess this more carefully. Of the previous studies that quantitatively investigated how basal insulin affects QoL in patients with T2D [11, 29, 30], most have used relatively broad, generic measures. One strength of our study is that we included a range of diabetes-specific PRO instruments to assess QoL, thus providing additional information on the specific issues that patients with T2D face, such as concerns related to hypoglycemia.

In the previous qualitative QoL study, patients who had switched to IDeg from another basal insulin reported greater energy and less fatigue, a heightened sense of security regarding hypoglycemia-related concerns, a reduced sense of diabetes as being burdensome, and enhanced feelings of adaptability and freedom [12]. The results in the current study support these findings. Patients who switched to IDeg from a previous basal insulin appeared to experience significantly improved well-being and feelings of freedom and flexibility. Specifically, patients reported they felt less pressured to eat snacks to avoid low blood glucose problems, felt less restricted about exercising, and felt they could be more spontaneous in their lives. Patients also reported significantly increased confidence and less anxiety around potential issues relating to hypoglycemia, such as safety when away from home or while driving. Patients also felt their diabetes became significantly less burdensome and experienced significantly less distress related to the day-to-day management of their diabetes.

In addition, sleep quality (hours of restful sleep) was also improved. Of note, sleep quality was associated with improved QoL on all of the other PRO domains assessed. Since factors such as improved glycemic control or reduced nocturnal glycemic variability could result in both improved sleep quality and in improved QoL, further studies to investigate these associations are warranted. One interesting area of future research would be to investigate the influence of basal insulin use on QoL, especially hypoglycemia outcomes and sleep quality, with more objective measures (e.g., actigraphy, polysomnography and continuous glucose monitoring).

Patients may have switched to IDeg due to dissatisfaction (their own and/or their healthcare providers') with their original insulin product. In that case, the observed QoL improvements may represent merely a regression to the mean, and therefore not resulting from any benefits accruing from the use of IDeg. To explore this issue, we conducted a set of secondary analyses to examine QoL improvement in that subset of patients (28.3% of the study sample) who were forced to change to IDeg due to insurance changes. We found that significant QoL changes were reported in this subset of patients, as well as in that subset who chose to switch, supporting a positive association between QoL and the use of IDeg.

Predictors of QoL improvements after switching to IDeg warrant further exploration. While it is understandable that a short experience with diabetes may be related to a more hopeful disposition on QoL in general, it is unclear whether cultural factors related to non-white ethnicity, for example, may contribute to improved QoL as well. We suspect that IDeg-associated QoL improvement may contribute to greater insulin adherence and persistence over time. Though this could not be evaluated in the current study, we hope to do so in future studies.

### Limitations

Only 2.4% of patients who were screened for eligibility met the inclusion criteria and completed the online survey. Further, the participants were selected from a convenience sample via a commercially available diabetes patient panel; therefore, their results may not be generalizable to the broader IDeg-using population due to potentially different demographic characteristics including a higher educational level and income, which could impact QoL. In addition, all data were self-reported by patients, including the diagnosis of T2D. However, the eligibility questionnaire asked multiple questions about the patient’s history and treatment of T2D, which increases our confidence that our study recruited an appropriate study population. Other clinical information such as previous basal insulin usage, T2D duration, and length of time using IDeg were also all self-reported. Other health conditions were not assessed in the survey, thus QoL could be confounded by the presence of comorbidities. Furthermore, the study was cross-sectional in nature; patients were not surveyed before and after starting IDeg but were asked to recall their perceptions thinking back to when they had been using their previous basal insulin, which likely introduced recall bias. Previous studies have found that when patients with insulin-treated T2D are asked to self-report the incidence of hypoglycemic events retrospectively, they underreport the incidence compared with when they are asked to self-report prospectively [31, 32]. Recall bias may be due to a number of factors, including the length of recall period [33]. In the present study, the fact that the majority of patients (64.5%) had switched to IDeg within a year, and nearly a third (32.9%) had switched to IDeg only within the last 3–6 months, may mitigate the length of the recall period as a source of recall bias.

## Conclusions

In this quantitative study, patients with T2D reported statistically significantly better QoL while using IDeg than while using their previous basal insulin across all the domains assessed. Study limitations include possible recall bias in cross-sectional, patient-reported data. The findings of our study highlight the specific domains and instruments that should be used in future prospective outcome studies to uncover positive responses that may have been missing in past studies. Significant self-reported improved scores after switching to IDeg included enhanced feelings of well-being, more confidence and less anxiety regarding potential issues with hypoglycemia, less diabetes burdensomeness, less regimen-related distress, greater feelings of freedom and flexibility in their daily lives, and more hours of restful sleep.

## Supplementary Information

Below is the link to the electronic supplementary material.

Supplementary file1 (DOCX 46 KB) Online Resource 1 Summary of survey structure and scoring schemes

Supplementary file 2 (DOCX 98 KB) Online resource 2 Percentage of patients who reported improved, unchanged, or decreased quality of life (N = 152)

## Data Availability

The datasets analyzed during the current study are available from the corresponding author on reasonable request.
