# Supplementary material for: Quality of life in patients with type 2 diabetes after switching to insulin degludec: results from a cross-sectional survey
Source: Qual Life Res. 2021 Feb 7;30(6):1629–40. doi: 10.1007/s11136-020-02753-6 (PMC8178133; doi:10.1007/s11136-020-02753-6)
Supplement: Supplementary file 1 — Supplementary file1 (DOCX 46 KB) Online Resource 1 Summary of survey structure and scoring schemes [file 11136_2020_2753_MOESM1_ESM.docx]

**Article title:** Quality of life in patients with type 2 diabetes after switching to insulin degludec: results from a cross-sectional survey

**Journal:** Quality of Life Research

**Authors:** Chioma Uzoigwe^1^ • Michael Radin^1^ • Carol M. Hamersky^1^ • Mitch DeKoven^2^ • Cassie Holt^3^ •

Swapna Karkare^4^ • William H. Polonsky^5,6^

**Authors’ affiliations:**

^1^ Novo Nordisk, Inc., Plainsboro, NJ, USA

^2^ IQVIA, Falls Church, VA, USA

^3^ IQVIA, New York, NY, USA

^4^ IQVIA, Deerfield, IL, USA

^5^ Behavioral Diabetes Institute, San Diego, CA, USA

^6^ University of California, San Diego, CA, USA

**Corresponding author:** Chioma Uzoigwe (coms@novonordisk.com)

**Online Resource 1** Summary of survey structure and scoring schemes

| **Section** | **Instrument/contents** | **Scale type and scoring scheme** |
| --- | --- | --- |
| Clinical history | Eight questions on clinical characteristics, including IDeg concentration, HbA1c level, and whether switching to IDeg was due to their insurance company no longer covering their previous basal insulin | – |
| Demographics | Four questions on employment status, highest educational level, type of health insurance, and marital/relationship status | – |
| Sense of physical well-being | Modified WHO-5. Patients rate five statements according to: a) how they felt on their previous insulin (directly before IDeg); and b) how they have been feeling over the past 2 weeks while using IDeg | Each item is rated on a six-level Likert scale ranging from 0 (“At no time”) to 5 (“All of the time”). Total raw score is the sum of the five items, and ranges from 0 (worst possible quality of life) to 25 (best possible quality of life) |
| Sense of safety | Modified HABS confidence and anxiety subscales. Patients indicate how much they agree or disagree with 10 statements: a) thinking back to the time on their previous insulin (directly before IDeg); and b) now, while using IDeg | Each item is rated on a five-level Likert scale ranging from 1 (“Strongly disagree”) to 5 (“Strongly agree”). Mean subscale score is the sum of the five items on that subscale divided by 5. Mean confidence subscale score ranges from 1 (lowest confidence) to 5 (highest confidence). Mean anxiety subscale score ranges from 1 (lowest anxiety) to 5 (highest anxiety) |
| Sense of diabetes as burdensome | Modified DDS emotional burden and regimen-related distress subscales. Patients rate 10 statements according to the degree to which the statement distressed or bothered them: a) thinking back to the time on their previous insulin (directly before IDeg); and b) during the past month, while using IDeg | Each item is rated on a six-level Likert scale ranging from 1 (“Not a problem”) to 6 (“A very serious problem”). Mean subscale score is the sum of the five items on that subscale divided by 5, and ranges from 1 (lowest distress) to 6 (highest distress) |
| Feelings of freedom and flexibility | Patients indicate the degree to which they agree or disagree with three statements on how the requirement to take insulin affects freedom and flexibility during daily life: a) thinking back to the time on their previous insulin (directly before IDeg); and b) during the past 4 weeks, while using IDeg | Each item is rated on a five-level Likert scale ranging from 1 (“Strongly agree”) to 5 (“Strongly disagree”). Mean score is the sum of the three items divided by three, and ranges from 1 (lowest degree of freedom and flexibility) to 5 (highest degree of freedom and flexibility) |
| Sleep quality | Patients state their average number of hours of restful sleep: a) thinking back to the time on their previous insulin (directly before IDeg); and b) during the past month, while using IDeg | Answer options are 0, 1, 2, 3, 4, 5, 6, 7, 8, 9, 10, or > 10 hours |

*DDS* Diabetes Distress Scale; *HABS* Hypoglycemia Attitudes and Behavior Scale; *HbA1c* glycated hemoglobin; *IDeg* insulin degludec; *WHO-5* World Health Organization (Five) Well-Being Index
