# Supplementary material for: Quality of life in patients with type 2 diabetes after switching to insulin degludec: results from a cross-sectional survey
Source: Qual Life Res. 2021 Feb 7;30(6):1629–40. doi: 10.1007/s11136-020-02753-6 (PMC8178133; doi:10.1007/s11136-020-02753-6)

**Article title:** Quality of life in patients with type 2 diabetes after switching to insulin degludec: results from a cross-sectional survey

**Journal:** The Patient – Patient-Centered Outcomes Research

**Authors:** Chioma Uzoigwe^1^ • Michael Radin^1^ • Carol M. Hamersky^1^ • Mitch DeKoven^2^ • Cassie Holt^3^ •
Swapna Karkare^4^ • William H. Polonsky^5,6^

**Authors’ affiliations:**

^1^ Novo Nordisk, Inc., Plainsboro, NJ, USA

^2^ IQVIA, Falls Church, VA, USA

^3^ IQVIA, New York, NY, USA

^4^ IQVIA, Deerfield, IL, USA

^5^ Behavioral Diabetes Institute, San Diego, CA, USA

^6^ University of California, San Diego, CA, USA

**Corresponding author:** Chioma Uzoigwe (coms@novonordisk.com)

**Online resource 2** Percentage of patients who reported improved, unchanged, or decreased quality of life (*N* = 152). *DDS* Diabetes Distress Scale; *HABS* Hypoglycemia Attitudes and Behavior Scale; *IDeg* insulin degludec; *WHO-5* World Health Organization (Five) Well-Being Index

Changes in quality of life were defined as the differences in scores for each measure/scale; improved quality of life = positive difference in score after switching to IDeg, no difference in quality of life = score before switching to IDeg and score after switching to IDeg was unchanged, decreased quality of life = negative difference in score after switching to IDeg.

For the WHO-5, a higher score indicates better quality of life. For the HABS measures, a higher score in the first domain indicates improved confidence, and a higher score in the second indicates heightened anxiety. For the DDS measures, a higher score indicates an increasing level of distress. For the three “feelings of freedom and flexibility” items, a higher score indicates living with a greater degree of freedom and fewer restrictions. Sleep quality was determined by the number of hours of restful sleep: increased number of hours after switching to IDeg = improved quality of life and decreased number of hours after switching to IDeg = decreased quality of life.


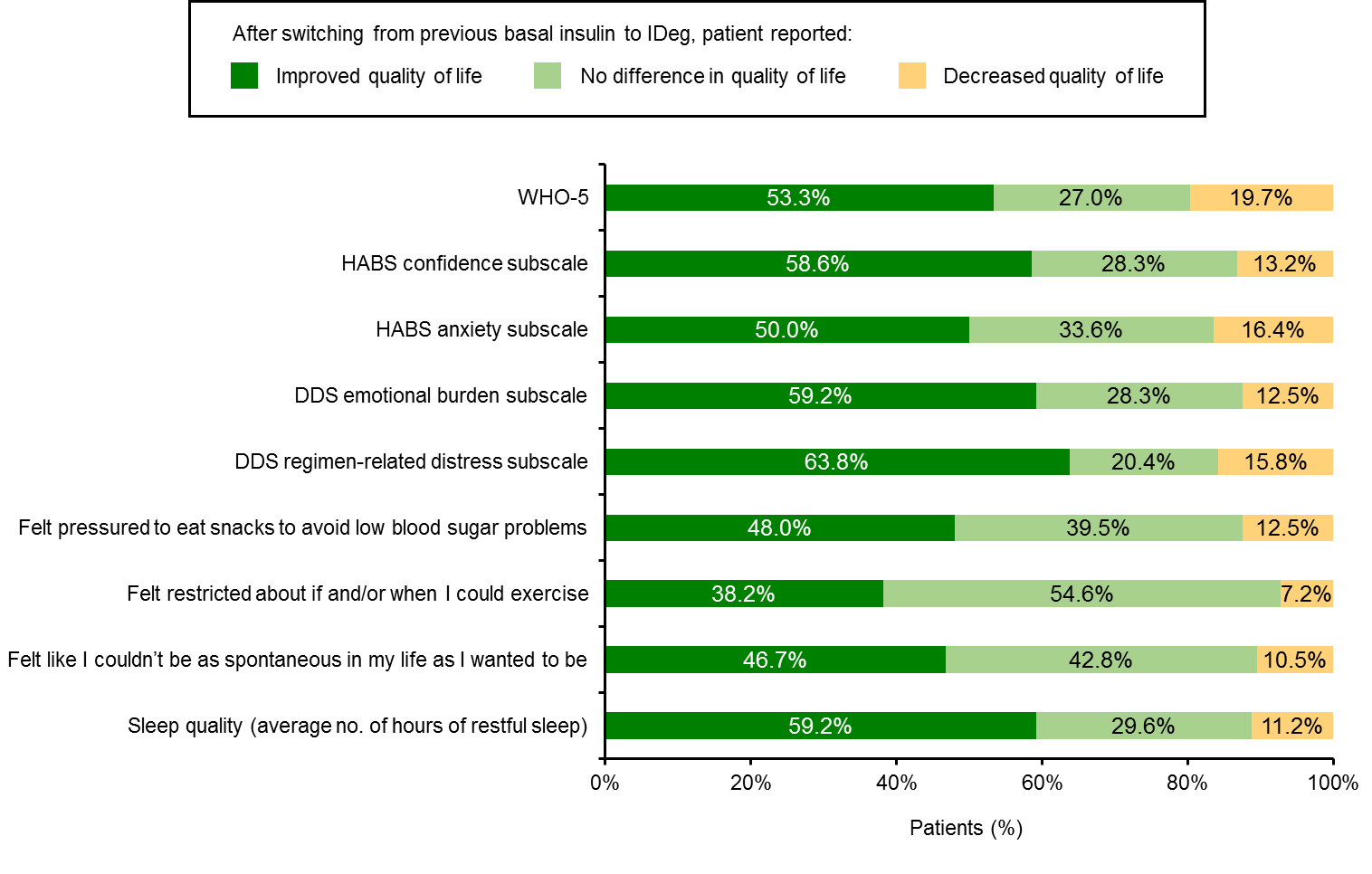

Supplement: Supplementary file 2 — (DOCX 98 KB) Online resource 2 Percentage of patients who reported improved, unchanged, or decreased quality of life (N = 152) [file 11136_2020_2753_MOESM2_ESM.docx]
